# The type I interferon system in the etiopathogenesis of autoimmune diseases

**DOI:** 10.3109/03009734.2011.624649

**Published:** 2011-10-29

**Authors:** Lars Rönnblom

**Affiliations:** Department of Medical Sciences, Section of Rheumatology, Uppsala University, Uppsala, Sweden

**Keywords:** Autoimmune, immune complex, interferon, plasmacytoid dendritic cell, systemic lupus erythematosus

## Abstract

Many patients with systemic autoimmune diseases have signs of a continuous production of type I interferon (IFN) and display an increased expression of IFN-α-regulated genes. The reason for the on-going IFN-α synthesis in these patients seems to be an activation of plasmacytoid dendritic cells (pDCs) by immune complexes (ICs), consisting of autoantibodies in combination with DNA or RNA-containing autoantigens. Such interferogenic ICs are internalized via the FcγRIIa expressed on pDCs, reach the endosome, and stimulate Toll-like receptor (TLR)-7 or -9, which subsequently leads to IFN-α gene transcription. Variants of genes involved in both the IFN-α synthesis and response have been linked to an increased risk to develop systemic lupus erythematosus (SLE) and other autoimmune diseases. Among these autoimmunity risk genes are IFN regulatory factor 5 (*IRF5*), which is involved in TLR signaling, and the signal transducer and activator of transcription 4 (*STAT4*) that interacts with the type I IFN receptor. Several other gene variants in the IFN signaling pathway also confer an increased risk to develop an autoimmune disease. The observations that IFN-α therapy can induce autoimmunity and that many autoimmune conditions have an on-going type I IFN production suggest that the type I IFN system has a pivotal role in the etiopathogenesis of these diseases. Possible mechanisms behind the dysregulated type IFNsystem in autoimmune diseases and how the IFN-α produced can contribute to the development of an autoimmune process will be reviewed.

## Introduction

The type I interferons (IFNs) are a family of related proteins, which were originally defined by their capacity to interfere with viral replication in cell cultures (1). This viral interference was the reason that the name ‘interferon’ was coined. Type I IFN is rapidly produced during viral invasion and, via inhibition of viral replication, constitutes our major defense system against viral infections. Type I IFN also have immunomodulatory functions which can best be described as a general activation of immune cells. For instance, type I IFN induces dendritic cell (DC) maturation and activation, with increased expression of MHC class I and II molecules, chemokines and chemokine receptors, as well as co-stimulatory molecules (2). The development of helper T cells along the Th1 pathway is promoted, and cytotoxic T cells are stimulated by type I IFNs (3,4). It can also cause B cell activation, differentiation, antibody production, and Ig isotype class switching (5,6). Thus, type I IFN is a potent immune adjuvant, and this observation has led to many clinical trials where type I IFN is administered to patients with both infectious and malignant diseases. In Uppsala, Professor Gunnar V. Alm at the Biomedical Centre set up a production of type I IFN for clinical use already in 1980. Buffy coats from blood centers in Sweden were used, and white blood cells were infected with Sendai virus for the induction of IFN production. Purified IFN was used mainly for patients with malignancies at the University Hospital in Uppsala, and beneficial effects were reported in several diseases (7–10). However, early on several colleagues in Uppsala noted an increased occurrence of autoantibodies and autoimmune disease during type I IFN treatment (11–13). These reports were the first indications of a causative role of type I IFN in human autoimmune diseases. We noted in a cohort of patients with malignant carcinoid tumors that as many as 19% of patients receiving long-term treatment with IFN-α eventually manifested an autoimmune disease (14), including systemic lupus erythematosus (SLE). Pre-existing autoantibodies were not necessary for development of autoimmunity, although presence of autoantibodies before IFN-α therapy considerably increased the risk for autoimmune disease. The conclusions from these observations are that type I IFN can both break tolerance and promote an on-going autoimmune reaction in man. The IFN-induced diseases also raised the question of the possible role of type I IFN in spontaneously occurring autoimmune diseases. This question has been intensively studied by several research groups during the last decade. In this review, the role of type I IFN in the etiopathogenesis of autoimmune diseases will be discussed with the focus on important discoveries by our research group. The potential application in clinical practice of our present knowledge of the type I IFN system will also briefly be mentioned.


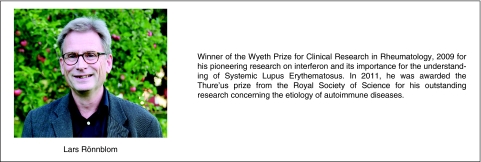


## The type I IFN system

There are 3 different types of IFNs (I–III), and among them the type I IFNs are the largest family that can be divided into 5 classes (IFN-α, -β, -ω, -ϵ, and -κ), of which IFN-α can be further divided into 12 subtypes encoded by 13 highly homologous genes clustered on chromosome 9. The type I IFN system is defined as the type I IFNs themselves and all inducers, cells, and molecules involved in the pathways leading to the production and effects of type I IFN. The type I IFN proteins bind to the same heterodimeric type I IFN receptor (IFNAR) consisting of two membrane-spanning polypeptide chains, IFNAR1 and IFNAR2 (15). Most types of cells can produce small amounts of type I IFN, but the principal type I IFN producer is the plasmacytoid dendritic cell (pDC), originally designated the natural IFN-producing cell and described in 1982–1983 (16–18). Plasmacytoid dendritic cells are infrequent but produce up to 10^9^ IFN-α molecules per cell within 24 h upon activation. The type I IFN genes are strictly regulated, and normally almost no constitutive IFN-α production can be detected in healthy individuals. Typically, the type I IFN production is induced by viruses, bacteria, or microbial nucleic acids when sensed by the pattern recognition receptors (PRRs), such as Toll-like receptors (TLRs), retinoic acid inducible gene 1 (RIG-I)-like receptors (RLRs), and nucleotide oligomerization domain (NOD)-like receptors (NLRs) (reviewed in (19)). Plasmacytoid dendritic cells express TLR7 and TLR9 in their endosomal membranes and can therefore become activated by pathogens that invade pDC through receptor-mediated endocytosis. The first step in the activation chain involves myeloid differentiation factor 88 (MyD88) that associates with a complex consisting of tumor necrosis factor receptor-associated factor 6 (TRAF6) and interleukin receptor associated kinase (IRAK) 1 and 4. These events lead to phosphorylation of IFN regulatory factor (IRF)-3, 5, and 7, translocation to the nucleus, and finally transcription of the type I IFN genes.

Ligation of the IFNAR by type I IFNs initiates several signal transduction pathways leading to expression of IFN-stimulated genes (ISGs). The classical IFNAR signaling pathway is the Jak-Stat pathway involving Janus family kinases, tyrosine kinase (Tyk) 2 and Jak1. The activated kinases recruit and phosphorylate the transcription factors Stat1 and Stat2, which finally associate with IRF9. The Stat1/Stat2/IRF9 complex translocates to the nucleus, binds to IFN-stimulated response elements (ISRE), and activates the transcription of hundreds of ISGs (20). The exact function of the majority of the ISG products is far from clear. However, some of the mechanisms conferring an antiviral state have been thoroughly investigated, showing that type I IFN interferes with several different steps in viral replication. The type I IFN induces and activates enzymes, such as myxovirus resistance MxA, 2'5'-oligoadenylate synthetase, and protein kinase (PKR16), which can inhibit viral transcription and translation and promote degradation of viral RNA (21).

Type I IFN also affects many key functions in the innate and acquired immunity as briefly described above. Several of these type I IFN effects promote the activation of immune cells and enhance an immune response. In addition, IFN-α can induce increased expression of autoantigens, such as Ro52 (22,23), and promote the release of autoantigens by induction of apoptosis (24), which in combination with an activation of the immune system can promote the development of an autoimmune reaction, if the IFN production is not properly controlled.

## SLE and other autoimmune rheumatic diseases

Compared to rheumatoid arthritis, SLE is a relatively rare disease, with an incidence of around 5 cases per 100,000 persons among Northern Europeans (25). There is a clear female preponderance (female to male ratio is 9:1), and most patients develop the disease between the ages of 15 and 50 years. SLE is regarded as the prototype autoimmune disease, and the reason is that a large number of different autoantibodies are produced in these patients and that most, if not all, cells in the immune system seem to be involved in the disease process (26). A prominent feature in SLE is an immune response to nucleic acid and associated proteins, which results in autoantibody production, immune complex (IC) formation, and organ inflammation. Before modern treatment was introduced, major organ involvement and/or infections were the most important causes of death. Today, cardiovascular diseases during late stage of SLE are one of the most challenging problems. For instance, in our SLE cohort a total of 10% of the patients are affected by stroke at a median age of 55 years.

Increased serum levels of IFN were described in SLE patients more than 30 years ago and initially suggested to be IFN-γ (27) but were later characterized as IFN-α (28). Further studies showed that serum IFN-α levels correlated to disease activity and to signs of immune activation, but also to several clinical disease manifestations (29). Early on, increased levels of IFN-α-induced proteins, such as 2'-5'oligoadenylate synthetase (30) and MxA (31), could also be demonstrated in the majority of SLE patients, confirming that bioactive type I IFN is produced in these patients. When genome-wide gene expression profiling became available, several research groups observed that a majority of SLE patients display an increased expression of type I IFN-regulated genes (an IFN signature), which is connected to a more severe clinical picture with nephritis or hematological manifestations (22,23,32,33). Pediatric SLE patients, who usually have a more severe disease compared to adult SLE patients, almost invariably display an IFN signature at early disease stages (22), which suggests that activation of the type I IFN system may be especially important in the initiation of the disease process.

Studies of other rheumatic conditions have demonstrated that several diseases show an IFN signature in both peripheral blood mononuclear cells (PBMC) and tissues from affected organs. Patients with primary Sjögren's syndrome have an IFN signature in specimens from salivary glands but also in PBMC (34,35). Similarly, this signature can be found in affected tissues and PBMC from patients with myositis, systemic sclerosis (SSc), and a subgroup of patients with RA (36). All these observations suggest a central and general role of the type I IFN system in the development of autoimmune rheumatic diseases. Because pDCs are the key players in the production of IFN-α, it seems logical to clarify their role in all these diseases.

## The plasmacytoid dendritic cell autoimmune diseases

In our early studies we noticed that the frequency of circulating pDCs is markedly reduced in SLE patients (37,38). However, functional studies of SLE pDC revealed that remaining single cells upon stimulation have a normal IFN-α-producing capacity. Several studies suggest that the reason for the decreased number of circulating pDCs seems to be a migration of these cells to tissues, because an increased number of pDCs can be detected in skin (39,40), lymph nodes (41), and renal tissue (42) from SLE patients. These pDCs are activated *in vivo* and synthesize IFN-α, which indicates that these cells in fact are responsible for the continuous IFN-α production seen in SLE patients. We could also demonstrate IFN-α-containing cells in salivary gland biopsies from patients with pSS (43) and myositis (44). Consequently, aberrant pDC activation may be an important step in the process that eventually leads to several different autoimmune diseases.

## Inducers of type I IFN production in autoimmune diseases

Normally, type I IFN synthesis is triggered by viruses, and the production is tightly regulated and limited in time. An important finding was therefore the observation that sera from SLE patients' IC have the capacity specifically to activate pDCs (45,46). Further studies revealed that such interferogenic ICs contain nucleic acids and are internalized via the FcγRIIa expressed on pDCs (47), reach the endosome, and stimulate the relevant TLR with subsequent activation of transcription factors and IFN-α production (48). This mechanism for induction of type I IFN production has been demonstrated *in vitro* for both DNA- and RNA-containing ICs. The nucleic acid-containing autoantigens in the interferogenic ICs can be generated from apoptotic or necrotic cells (49), which is relevant given the increased apoptosis and reduced clearance of apoptotic cells in SLE (50,51). Recent studies have shown that neutrophils undergoing so-called NETosis also have the capacity to provide interferogenic autoantigens (52,53), demonstrating that several pathways can lead to pDC activation in SLE. The complement component C1q has the capacity to decrease the IFN-α production by interferogenic ICs (54,55), and this effect may at least partially explain the increased incidence of SLE in C1q-deficient individuals (56).

ICs containing both DNA and RNA have the capacity to activate pDCs, but RNA-containing ICs (RNA-IC) that trigger TLR7 seem to be especially potent as IFN-α inducers (57,58). Among these are ICs generated by autoantibodies against snRNP or SSA in combination with the appropriate autoantigen. There is in SLE patients a correlation between serum IFN-α activity and presence of autoantibodies to RNA-binding proteins (59). Since some of these autoantibodies appear several years before the appearance of clinically overt SLE disease (60) and show cross-reactivity with viral epitopes (61), the initial trigger for the production of antibodies with IFN-α-inducing capacity could well be a viral infection. This scenario would connect viral infections with the generation of interferogenic ICs, which partly could explain the long-sought connection between infectious diseases and autoimmunity.

It is important to notice that ICs with the capacity to trigger pDC to IFN-α production can be generated by autoantibodies from patients with all diseases displaying an interferon signature (43,44,62). So, although it remains to be shown whether interferogenic ICs in fact are responsible for the on-going type I IFN production in these diseases *in vivo*, our data suggest that this may well be the case.

## The regulation of type I IFN response in autoimmunity

An obvious question is why the type I IFN system is not down-regulated in patients with autoimmune disease, because both type I IFN production and autoantibody formation regularly occur during infections. An important finding was the observation that pDCs are regulated by other immune cells and that natural killer (NK) cells enhance the IFN-α response by pDC stimulated with RNA-IC (63). In contrast, monocytes have a strong inhibitory effect on the NK cells, which is mediated via prostaglandin E2, TNF-α, or reactive oxygen species. Interestingly, this inhibitory function by monocytes is diminished in SLE patients (63), which may contribute to the continuous activation of the type I IFN system. Recently, we have shown that NK cells enhance the pDC response to RNA-IC via MIP-1β and LFA-1, and that the pDC-NK cross-talk in SLE is promoted by IL-12 and IL-18 (64). Much less is known about the role of the adaptive immune system on the pDC function, but this is an important area for further studies. However, it is clear that if we understood the regulation of the type I IFN system, we could develop more powerful therapeutic strategies.

## Gene variants in the type I IFN system and risk for autoimmunity

**Figure 1.**
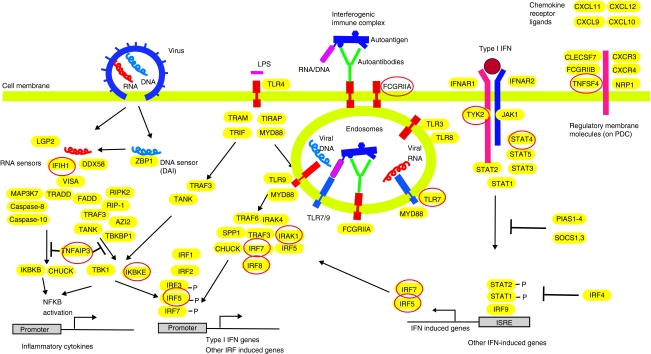
Genes connected to the type I interferon production and response in pDC. *Left:* Genes involved in the response to viral RNA/DNA by the cytosolic pattern recognition receptors leading to transcription of type I IFN genes. Via NF-kB activation, genes for several inflammatory cytokines are also activated. TNAIP3 is involved in the down-regulation of a pro-inflammatory response. *Middle:* Induction of IFN production by interferogenic DNA/RNA-containing immune complexes (IC) as outlined in the text. TLR3 is expressed by many different cell types and can be activated by viral RNA, while bacterial LPS is recognized via TLR4 that signals via two different pathways. *Right:* IFN signaling via the type I IFN receptor (IFNAR). The interferon-stimulated response elements (ISREs) induce expression of several hundreds of IFN-induced genes, including IRF5 and IRF7. The pDC response is modulated by several chemokines. Variants of genes in red circles are associated to an increased risk for SLE.

Among the many identified risk genes for SLE, a large number are connected to pathways that are involved in the IFN-α production or sensitivity (Figure 1). The transcription factor IRF5, which is constitutively expressed in pDC (65), was the first identified gene directly involved in IFN-α gene activation that was associated to increased risk for SLE (66). Subsequent studies have shown that polymorphisms in *IRF5* are important for the susceptibility to several other autoimmune diseases, including rheumatoid arthritis (67) and pSS (68). Recently, the allele variants with the highest probability of being causal in SLE were identified and shown to affect the *IRF5* expression, which is increased in PBMC from SLE patients (69). We have shown that alternative splicing of *IRF5* is significantly up-regulated in PBMC from SLE patients and that a risk haplotype is associated with the enhanced *IRF5* transcript and protein expression (70). An *IRF5* risk haplotype is associated to a high serum IFN-α activity in patients and especially in those with autoantibodies to RNA-binding proteins or double-stranded DNA (71), linking SLE genetic susceptibility to the presence of interferogenic ICs. Recently, we found that also a gene variant of IKBKE (IKK-ϵ), which is a central signal-transducing molecule for the cytosolic RNA/DNA sensors and TLR4, is associated with SLE (72).

Among gene products involved in the response to IFN-α, the STAT4 that interacts with the cytoplasmic part of the IFNAR (73) is strongly associated to SLE (74). In SLE patients there is an association between *STAT4* genotype and a more severe phenotype, which includes nephritis and presence of anti-dsDNA autoantibodies (75,76). Polymorphisms in the Janus kinase *TYK2*, which binds to IFNAR and is required for signaling through this receptor, are also associated to SLE (66,77). These data provide further evidence for a link between the IFN-α response and the disease process in SLE. Additional susceptibility genes for SLE can be involved in the activation of the type I IFN system by other mechanisms, for instance via generation of autoantigens that contain nucleic acid or increased production of autoantibodies causing the formation of increased levels of interferogenic ICs (78).

## The connection between the type I IFN system and disease manifestation

Several observations suggest that IFN-α may play an important role in some of the clinical manifestations in SLE patients. There is an association between increased serum levels of IFN-α and fever, skin rash, and leukopenia (29), which perhaps is not surprising considering that these symptoms are commonly seen during viral infections. The IFN-regulated chemokines are connected to organ damage, which indirectly supports a role for type I IFN in disease outcome (79). Among the more specific SLE manifestations, an early study observed that patients with lupus psychosis have detectable levels of IFN-α in the cerebrospinal fluid (CSF) (80), which is intriguing given the observed neuropsychiatric adverse effects during IFN-α treatment (81). Recently, autoantibodies with the ability to form very potent interferogenic ICs together with RNA-containing autoantigens were demonstrated in the CSF of SLE patients with neuropsychiatric manifestations (82). These ICs also induced other chemokines and pro-inflammatory cytokines of possible relevance for the CNS manifestations frequently seen in SLE, which indicates that the interferogenic ICs may be directly involved in CNS lupus.

Results from an experimental model suggest that IFN-α can drive the nephritis and end- organ damage in SLE (83), and it has been shown that pDCs accumulate in active human SLE nephritis (42). Major organ involvement in SLE patients, such as nephritis, is also connected to a more pronounced IFN-α signature (23), and IFN-α-regulated genes are over-expressed in the glomeruli from SLE nephritis (84). The role of IFN-α in the premature atherosclerosis typically seen in SLE patients is unclear, but the type I IFN system may contribute to the atherosclerotic process by several mechanisms (85). For instance, IFN-α may impair endothelial cell differentiation (86) and affect platelet function (87), but also promote foam cell formation (88). Furthermore, pDCs are present in atherosclerotic plaques from carotid lesions where they can act, via the IFN-α produced, as inflammatory amplifiers and destabilize the plaque (89).

There is also a possibility that genes within the type I IFN signaling pathway can be used as markers for organ involvement and severity. Thus, there is an association between STAT4 genotype and risk for a more severe disease phenotype that includes nephritis (75,76) and stroke (90). The latter observation is interesting, because the association between a *STAT4* risk variant and stroke was of the same magnitude as the association between stroke and hypertension, indicating that autoimmune processes may be very important for many disease manifestations in SLE, even those that traditionally are not regarded as autoimmune. Furthermore, a combination of risk alleles within the type I IFN signaling pathway dramatically increases the risk for SLE (76), which illustrates how genetic mapping in the future perhaps could aid in the prediction of risk for disease.

## The role of the type I IFN system in the development of autoimmunity

**Figure 2.**
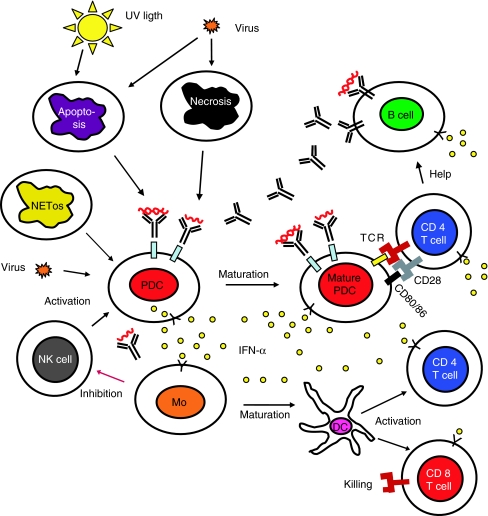
The role of the type I interferon system in the etiopathogenesis of systemic autoimmune diseases. A viral infection induces IFN-α production in pDC and the release of autoantigens from dying cells. The produced IFN-α activates both the innate and adaptive immune system as described in the text. In individuals with a genetic set-up that causes a strong IFN-α production and/or a marked IFN-α response, tolerance is broken, and antibodies against nucleic acid-containing autoantigens are produced. These antibodies together with the autoantigens form interferogenic ICs that stimulate the pDC to IFN-α synthesis and the B cells to increased autoantibody production, which causes a vicious circle with a continuous IFN-α production and an on-going autoimmune reaction. NK cells promote the IFN-α production and activated monocytes down-regulate the NK cells, but this latter function seems to be deficient in lupus. Figure modified from (101). (DC = dendritic cell; IC = immune complex; IFN = interferon; Mo = monocyte; NK = natural killer; pDC = plasmacytoid dendritic cell; TCR = T cell receptor).

The many findings concerning the type I IFN system in SLE patients have been put together into an etiopathogenic model of SLE, which also includes other observations in this disease (Figure 2). This model was first described by us in 1999 (45) and has subsequently been updated (39,91). It is envisioned that an initial infection by a virus induces type I IFN production and release of cellular material from dying cells. The extracellular autoantigens from apoptotic and necrotic cells as well as NETs from granulocytes then trigger B cells to autoantibody production against RNA and DNA-binding proteins in individuals prone to autoimmune reactions. ICs will be formed, which act as endogenous type I IFN inducers that cause a prolonged stimulation of type I IFN production by pDC. The excessive release of endogenous DNA/RNA in combination with impaired clearance of apoptotic cell material will facilitate the generation of interferogenic IC. Several drugs and environmental factors can contribute to the generation of autoantigens that can form more ICs. The type I IFN produced activates B cell differentiation, Ig switch, and generation of antibody-producing plasma cells as well as long-lived memory cells. Estrogens will further aid in the activation of autoreactive B cells. IFN-α will cause increased exposure of autoantigens such as Ro52 and prime for enhanced IFN-α production by pDC. As a consequence, conventional DCs and macrophages up-regulate their expression of co-stimulatory molecules and become more effective in their antigen presentation. In addition, type I IFNs prolong the survival of activated T and B lymphocytes and suppress regulatory T cells. Taken together a chronic activation of the type I IFN system will occur and drive an autoimmune process leading to chronic inflammation and tissue damage in a vicious circle manner.

## IFN-α as a therapeutic target

The development of therapies aiming to inhibit type I IFN production in autoimmune diseases has been stimulated by the observation that type I IFNAR knock-out murine lupus models have a reduced disease activity (92,93). Results from the first phase I clinical trial using a single injection of anti-IFN-α monoclonal antibodies in SLE patients were recently reported (94,95). The anti-IFN-α treatment caused a dose-dependent inhibition of type I IFN inducible genes in both peripheral blood and skin biopsies, as well as a reduction in clinical disease activity. In addition, GM-CSF, TNF-α, IL-10, and IL-1β inducible gene signatures, as well as BAFF mRNA expression, were neutralized in some patients (96), demonstrating the close interaction between the type I IFN system and other pro-inflammatory pathways. The observation that a single injection of an anti-IFN-α antibody could give a sustained neutralization of the IFN signature is of particular interest and supports the view that the on-going production of IFN-α in SLE is at least partly a result of a self-perpetuating vicious circle (97). So far, no increase in serious viral infections has been reported among anti-IFN-α-treated patients, which could be due to the fact that, besides IFN-α, several other type I IFNs exist with strong antiviral activity (98). Whether these latter type I IFNs are sufficiently potent to protect anti-IFN-α-treated SLE patients from serious complications during for instance a flu pandemic remains to be established, and this can only be addressed in larger clinical trials. Other possible therapeutic targets exist within the type I IFN system, such as the type I IFN receptor, the BDCA-2 antigen on pDC (38,99), or oligodeoxyribonucleotide or oligoribonucleotide TLR antagonists (100). None of these agents have been tested so far in human SLE patients.

## Conclusion

The type I IFN system is activated in patients with several systemic autoimmune diseases, which seems to be of major importance in the disease process. The genetic and immunological background to the increased production of IFN-α is to some extent clarified, but several questions still remain to be answered. Among these are the mechanisms that lead to different disease phenotypes, despite a similar IFN signature, and the optimal way to down- regulate the type I IFN system. Despite this, a number of studies that target the increased IFN-α levels in several autoimmune diseases are in an early clinical phase.
